# 

**Published:** 1883-03

**Authors:** 


### CONTENTS.

### COMMUNICATIONS.

On Certain Conditions of Dead Teeth.......... 105

The Radical Cure of Aveolar Abscess.......... 111

Erosion of the Teeth Considered as a Retrospcction Sign of Infantile Eclampsia.......... 117

### SELECTIONS.

Extracts from Medical Clinic of Prof. Jas. T. Whittaker.......... 129
Case of Deafness Probably Caused by Amalgam Fillings.......... 133
Chloroform Water.......... 134
Disinfection by Hot Air.......... 135
The Late Dr. Marshall H. Webb.......... 135
Loss of Both Parotid Glands.......... 136
Female Brain Work.......... 137
Office Lights.......... 138
Proceedings.......... 139
Central Illinois Dental Society.......... 144
International Congress.......... 145
Correspondence.......... 146

### EDITORIAL.

Michigan State Dental Society.......... 148
Obituary.......... 149
Annual Commencement of the Dental Department of the University of Maryland.......... 149

